# IL-38 restrains inflammatory response of collagen-induced arthritis in rats via SIRT1/HIF-1α signaling pathway

**DOI:** 10.1042/BSR20182431

**Published:** 2020-05-28

**Authors:** Bing Pei, Keyan Chen, Shenglai Zhou, Dongyu Min, Weiguo Xiao

**Affiliations:** 1 Department of Rheumatology and Immunology, The First Affiliated Hospital, China Medical University, China; 2 People’s Hospital of China Medical University, China; 3 Department of Laboratory Animal Science, China Medical University, China; 4 Experimental Center of Traditional Chinese Medicine, the Affiliated Hospital of Liaoning University of Traditional Chinese Medicine, China

**Keywords:** CIA, IL-38, inflammatory response, SIRT1/HIF-1α signaling pathway

## Abstract

**Objective:** To observe the restraining effect of IL-38 on inflammatory response in collagen-induced arthritis rats (CIA), and to explore the regulatory mechanism of SIRT1/HIF-1α signaling pathway.

**Methods:** 40 SD rats were randomly divided into Control group, CIA group, CLL group and CLH group, with 10 rats in each group; CIA rat model was established. The effects of IL-38 on arthritis index, inflammatory response, osteogenic factor and angiogenic factor were observed by methods including HE staining, ELISA, immunohistochemical and immunofluorescence. Human synoviocytes were cultured *in vitro*, and SIRT1 inhibitors were added to detect the expression for relating factors of SIRT1/HIF-1α signaling pathway by Western blot.

**Results:** IL-38 could alleviate CIA joint damage and restrain inflammatory response, could up-regulate the expression of OPG in CIA rats and could down-regulate the expression of RANKL and RANK. IL-38 could restrain the expression of VEGF, VEGFR1, VEGFR2 and HIF. Moreover, we found that IL-38 could up-regulate the SIRT1 expression and down-regulate the HIF-1α, TLR4 and NF-KB p65 expression in CLL and CLH groups. From the treatment of synoviocytes to simulate the CIA model and the treatment of SIRT1 inhibitors, we demonstrated that the inhibitory effect of IL-38 on inflammatory factors and regulation of SIRT1/HIF-1α signaling pathway-related proteins were inhibited.

**Conclusion:** IL-38 can restrain the inflammatory response of CIA rats, can promote the expression of osteogenic factors, can inhibit neovascularization, and can alleviate joint damage in rats. The mechanism may be related to the regulation of SIRT1/HIF-1α signaling pathway.

## Background

Rheumatoid arthritis (RA) is a systemic immune system disease characterized by hyperplasia of chronic synovitis and formation of pannus without a clear etiology [1]. Neovascularization is accompanied by synovial hyperplasia and inflammatory cell infiltration, which is the basis of vasospasm formation and joint destruction [2]. This inflammatory process is regulated by cytokines, thus the targeted therapy against cytokines has become an effective treatment for RA.

RA is related with the chronic production of proinflammatory cytokines. IL-38, an important proinflammatory cytokine, is a new member of the IL-1 family with receptor antagonist activity, which has a restraining effect on inflammatory immune processes [3]. IL-38 can inhibit the production of IL-17 and IL-22 by peripheral blood mononuclear cells stimulated by candida albicans, which plays a significant role in various autoimmune diseases [4]. The polymorphism of *IL-38* was reported to be associated with rheumatoid arthritis [5]. Moreover, IL-38 levels in RA patients’ serum were higher than those in healthy control groups, and IL-38 expression in synovial membranes of RA patients and arthritis rats was increased, relating with IL-1β, CCL-3, CCL4 and M-CSF [6]. Therefore, IL-38 is considered to have an inhibitory effect in the pathological process of rheumatoid arthritis.

RA is also associated with low oxygen tension, as deficit vascular capacity could not fulfill the oxygen demands of synovial hyperplasia in progressive RA. HIF-1 is a transcriptionally active nuclear protein, which could participate in the hypoxia adaptation, the inflammation development and tumor growth [7–9]. SIRT1 is a histone deacetylase dependent on nicotinamide adenine dinucleotide, which plays an important role in cell stress, inflammation, genomic stability and apoptosis [10–13]. SIRT1 was reported to regulate the development of rheumatoid arthritis through interaction with HIF-1 [14]. HIF-1 could be induced by TNF-α in synovial cells, leading to chronic inflammation by promoting the production of inflammatory cytokines and inhibiting apoptosis [15]. Therefore, we aim to establish the CIA model by collagen induction method and observe the inhibitory effect of IL-38 on CIA inflammatory response.

Moreover, the detailed regulatory mechanism of SIRT1/HIF-1α signaling pathway in CIA model was further investigated

## Subjects/Animals and methods

### Experimental animals and group assignment

Forty SPF SD rats weighing 200–220 g were purchased from Beijing Weitong Lihua Experimental Animal Technology Co., Ltd., and were randomly divided into normal control group (control group, *n* = 10), CIA model group (CIA group, *n* = 10), CIA+IL-38 (1 ng/g/d) group (CLL group, *n* = 10) and CIA+IL-38 (5 ng/g/d) group (CLH group, *n* = 10). The experimental animals were kept in the experimental animal department of China Medical University. The animal diet was normal and the light was exchanged for 12 h. The test passed the ethical review of experimental animal welfare in China Medical University (IACUC NO.2018102).

### CIA animal model establishment

CIA animal model was prepared as described by Trentham et al [16]. The rats were anesthesia via abdominal injection of 35 mg/kg 1% pentobarbital sodium. Then, the 25 mg/ml emulsion was formed by the combination of 500 mg of bovine type II collagen (Chondrex Inc., Washington, U.S.A.) in 1 ml of an emulsion in a 0.3% (Cosmo Bio., Tokyo, Japan) acetic acid solution and 500 mg of Freund’s incomplete adjuvant (Chondrex Inc., Washington, U.S.A.), which was intradermally injected at multiple points on the root of rat’s tail. And the second booster was performed, as 0.5 ml of the same emulsion was intracutaneously injected into the Lt. plantar surface of rats after 3 weeks.

Rats in the CLL group and the CLH group were respectively injected recombinant murine IL-38 intravenously (iv) daily at the base of the tail 1 and 5 ng/g/d for 10 days from the 24th day after the initial immunization, and the control group was injected with the same amount of physiological saline. The skin color, skin temperature, skin infection status of the hind paw and the joints of the rats in each group were observed continuously, and the hind foot movement and the swelling of the joints were observed.

### Rat rheumatoid arthritis score

After continuous observing the hind paw condition of the rats, the joint symptom scores were evaluated by the arthritis index integration method according to the degree, extent and deformation of the joint redness as well as the symptoms of the loss of luster and the decreased activity of the hair. The two hind paws of the rats were quantitatively scored and the average arthritis scores of each group were calculated according to the joint symptom score of each rat. The joint symptom scores were as follows: arthritis was divided into 5 grades: 0 points – no redness; 1 point – red spots or mild swelling; 2 points – moderate swelling of the joints; 3 points – severe swelling; 4 points – joint deformation without the ability to bear weight. The total score for arthritis is 16 points.

### HE staining

Rats in each group were over-anesthetized to be killed after modeling for 35 days, and implemented euthanasia and extraction of articular decalcification solution. After 1 month, the alcohol was dehydrated, and paraffin-embedded wax was prepared. Four-micrometer sections were cut and dewaxed as well as stained with hematoxylin for 5 min; the sections were washed with PBS, and 1% hydrochloric acid alcohol was differentiated and stained with eosin for 30 s. After routine dehydration and transparent treatment, the neutral gum was sealed and the pathological changes of brain tissue were observed under light microscope.

### ELISA

Osteogenic factor in serum including OPG (SEA108Ra, USCN, Wuhan, China), RANKL (SEA855Ra, USCN, Wuhan, China) and RANK (SEC057Ra, USCN, Wuhan, China) were selected by ELISA kit; Inflammatory factors in serum and supernatant including TNF-α (SEA133Ra, USCN, Wuhan, China), IL-1β (SCA563Ra, USCN, Wuhan, China), IL-6 (SEA079Ra, USCN, Wuhan, China), IL-10 (SEA056Ra, USCN, Wuhan, China), IL-17 (SEA063Ra, USCN, Wuhan, China) and IL-13 (SEA060Ra, USCN, Wuhan, China) were detected, and the operation steps are carried out according to the instruction manual of the kit: after the kit temperature is equilibrated to room standard (20–25°C), the required reaction plate is taken out, and 100 µl of standard product and 100 µl of the diluted sample are successively added to the corresponding reaction plate. After gently shaking for 30 s and mixing evenly, incubating it for 20 min in the environment with 20–25°C; washing the reaction plate with a washing machine, adding 100 µl serum sample to each hole, and incubating for 2 h at the 37°C; washing the plate and adding 100 µl HRP mark to each hole, and incubating for 30 min at the 37°C. Washing the plate, adding coloring solution A and coloring solution B 50 µl respectively, and darking color for 15 min; adding 50 µl of stop solution, microplate reader (EXL808, U.S.A.) reading 450 nm OD value; drawing the standard curve with the OD value on the ordinate and the standard product on the abscissa. The concentration value corresponding to the sample is obtained according to the curve equation.

### Immunohistochemical

Dewaxing to water, the slice soaked in 3% hydrogen peroxide solution after 15 min, PBS cleaning section, 0.1 M sodium citrate solution for antigen after repair, add VEGF (dilution as 1:100, Abcam, ab53465), VEGFR1 (dilution as 1:250, Abcam, ab32152), VEGFR2 (dilution as 1:100, Abcam, ab2349) antibody, 4°C incubation overnight, PBS cleaning slice, add 2 resistance of biotin labeling, 37°C after incubation for 30 min, PBS cleaning slice, join the DAB chromogenic liquid color, hematoxylin dyeing redyeing nuclei, neutral resin sealing film, were observed under microscope.

### Immunofluorescence

Immunofluorescence was used to detect articular angiogenesis, osteogenesis and SIRT1/HIF-1α signaling pathway-related proteins in CIA rats. The wax pieces prepared in the previous stage (with the 8-μm thick) were dried in cold air and blocked at room temperature for 20 min with the serum. After drying, the pieces were added with primary antibody, SIRT1 (Abeam, ab110304), HIF-1α (Abcam, ab92498), TLR4 (Abeam), ab22048), NF-κB p65 (Abcam, ab16502) and were incubated overnight at 4°C After washing well with PBS, adding HRP-labeled goat anti-rabbit IgG (Abeam, ab6721) and were incubated for 40 min, washed well with PBS; slice was added dropwise in the circle after slightly drying DAPI staining solution, and incubated for 10 min at room temperature in the dark; the slides were washed in PBS on a decolorizing shaker for three times with 5 min each time; the sections were slightly dried and sealed with anti-fluorescence quenching tablets; Photographing: The sections were observed under a fluorescence microscope and images were taken.

### Human synovial cells (HS)

The synovial tissue of RA patients undergoing arthroplasty was taken and primary synovial cells were cultured, while the normal human synovial cells (HS) were taken as the control. The cells were cultivated in RPMI-1640 (Gibco Life Technologies, Grand Island, NY, U.S.A.) with supplemented 10% fetal calf serum (FBS; HyClone, Logan, UT, U.S.A.) at 37°C in a 5% CO_2_ incubator. The cells were divided into control group (Control group), induced arthritis model group (CLA group); IL-38+ induced arthritis model group (IL-38 group); IL-38+ SIRT1 inhibitor + model group (SIRT1 group).

### Western blot

After collecting the cell and tissue homogenate, add to the RIPA (Thermo, 89900) lysate containing protease inhibitor, lyse on ice for 30 min, collect the supernatant, and concentrate the concentration of the collected protein using BCA (Thermo, 23225) protein quantification kit. The concentration of the solution was measured, and the protein was electrophoresed by SDS-PAGE, and OPG (Abcam, ab73400), RANKL (Abcam, ab100749), RANK (Abcam, ab13918), VEGF (Abcam, ab32152), VEGFR1 (Abcam, ab32152), VEGFR2 (Abcam, ab32152), HIF (Abcam, ab2185), SIRT1 (Abcam, ab110304), HIF-1α (Abcam, ab187524), TLR4 (Abcam, ab22048), NF-KB (Abcam, ab16502) antibody were added. Incubate at 4°C overnight, wash the PVDF membrane with PBS, add horseradish peroxidase-labeled secondary antibody, incubate for 2 h at room temperature, and color the protein using ECL luminescence kit and gel imaging system. The results were analyzed by absorbance using ImageJ.

### qRT-PCR

The collected cells and the fully ground brain tissue were added to a solution of Trizol (Invitrogen, 15596026), and the total RNA in the tissues and cells were separated and extracted according to the Trizol reagent operating instructions. After the first strand cDNA (Thermo, K1622) was synthesized by reverse transcription, detect the reaction of OPG, RANKL, RANK according to the qRT-PCR (Qiagen, 204057) method kit instructions. The primer sequence is shown in Table 1, synthesized by Shanghai Shenggong Biotech Co., Ltd. The reaction conditions were pre-denaturation: 95°C for 30 s; PCR reaction: 95°C for 5 s, 60°C for 20 s, 40 cycles; melting curve analysis: 95°C for 1 s, 65°C for 15 s, 95°C for 5 s. After the reaction was completed, the amplification curve and the melting curve were confirmed.

**Table 1:** Primer sequences

Gene name	Direction	Oligonucleotide sequences
GAPDH	Forward	TTCAACGGCACAGTCAAG
	Reverse	TACTCAGCACCAGCATCA
OPG	Forward	CAGAGGACCACAATGAACA
	Reverse	TTAGGTAGGTGCCAGGAG
RANKL	Forward	CATCGCTCTGTTCCTGTA
	Reverse	TTCTGTGTCTTCGCTCTC
RANK	Forward	GGACGACGGAATCAGATG
	Reverse	CCACCACTACCACAGAGA

### Statistical analysis

Statistical analysis was performed by using SPSS 19.0 (IBM, California, U.S.A.). The results were expressed as mean ± standard deviation and ANOVA variance analysis was performed for intergroup comparisons followed by the Tukey procedure. The *t*-test was used for pairwise comparison while the analysis of variance was used for comparison between groups. Differences in pathology and immunostaining between groups were determined using the Fisher’s test A value of *P*<0.05 was considered statistically significant.

## Results

### IL-38 improves joint damage in CIA rats

**Figure 1:**
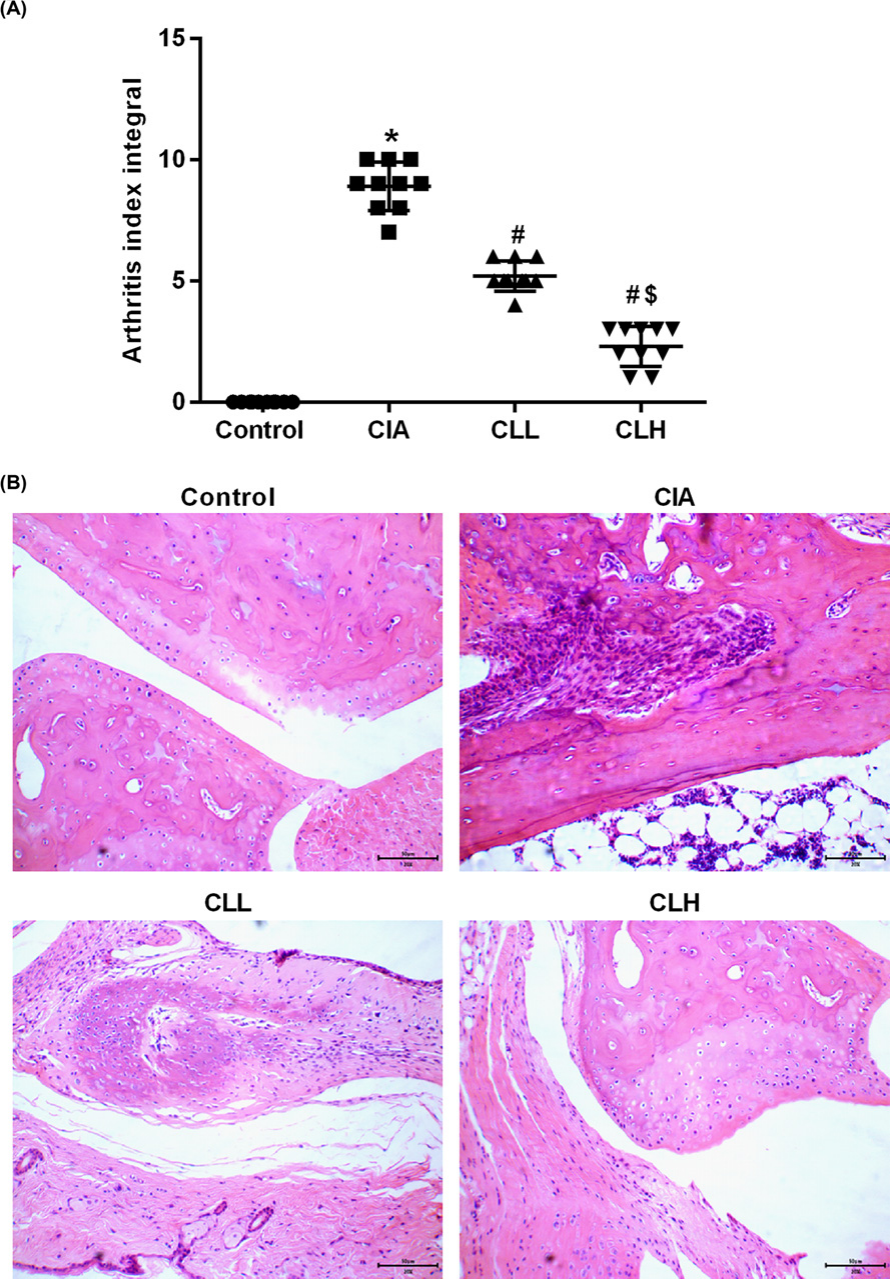
IL-38 improves joint damage in CIA rats Arthritis index and HE staining were used to observe the CIA joint damage. (**A**) Joint symptom scores; (**B**) HE staining. * vs control group, *P*<0.05; # vs CIA group, *P*<0.05; $ vs CLL group, *P*<0.05.

After constructing the CIA rat model for 14 days, the foot and the double stepped joint began to show acute inflammation including redness and heat, use joint symptom scores to show the degree of joint damage. Compare with control group, the toe and the double stepped joint were obviously swollen in CIA group. After giving different concentrations of IL-38, the degree of swelling of the foot extension and the double step joint was alleviated, and the degree of remission of the high dose was better than the low (Figure 1A).

The pathological changes of the joint area of rats were further observed by HE staining, and the ankle joint structure of the control group was intact without hyperplasia.

Furthermore, there was no inflammatory cell infiltration in the interstitial, the articular cartilage surface was smooth and flat, and the chondrocytes showed no hyperplasia. In the CIA group, the ankle joint structure was disordered, the synovial cells proliferated obviously, and a large number of inflammatory cells infiltrated in the interstitial; the vascular was formed, and some even eroded to the cartilage surface to destroy the joint. In the CLL and the CLH group, the proliferation of synovial cells of the ankle joint was not significant, and a small number of inflammatory cells were infiltrated in the interstitial. The cartilage showed focal degeneration at different degrees, and the cells in the cartilage lacuna were absent (Figure 1B). It can be seen that IL-38 can alleviate joint damage in rats with collagen-induced arthritis.

### IL-38 inhibits inflammatory response in CIA rats

**Figure 2:**
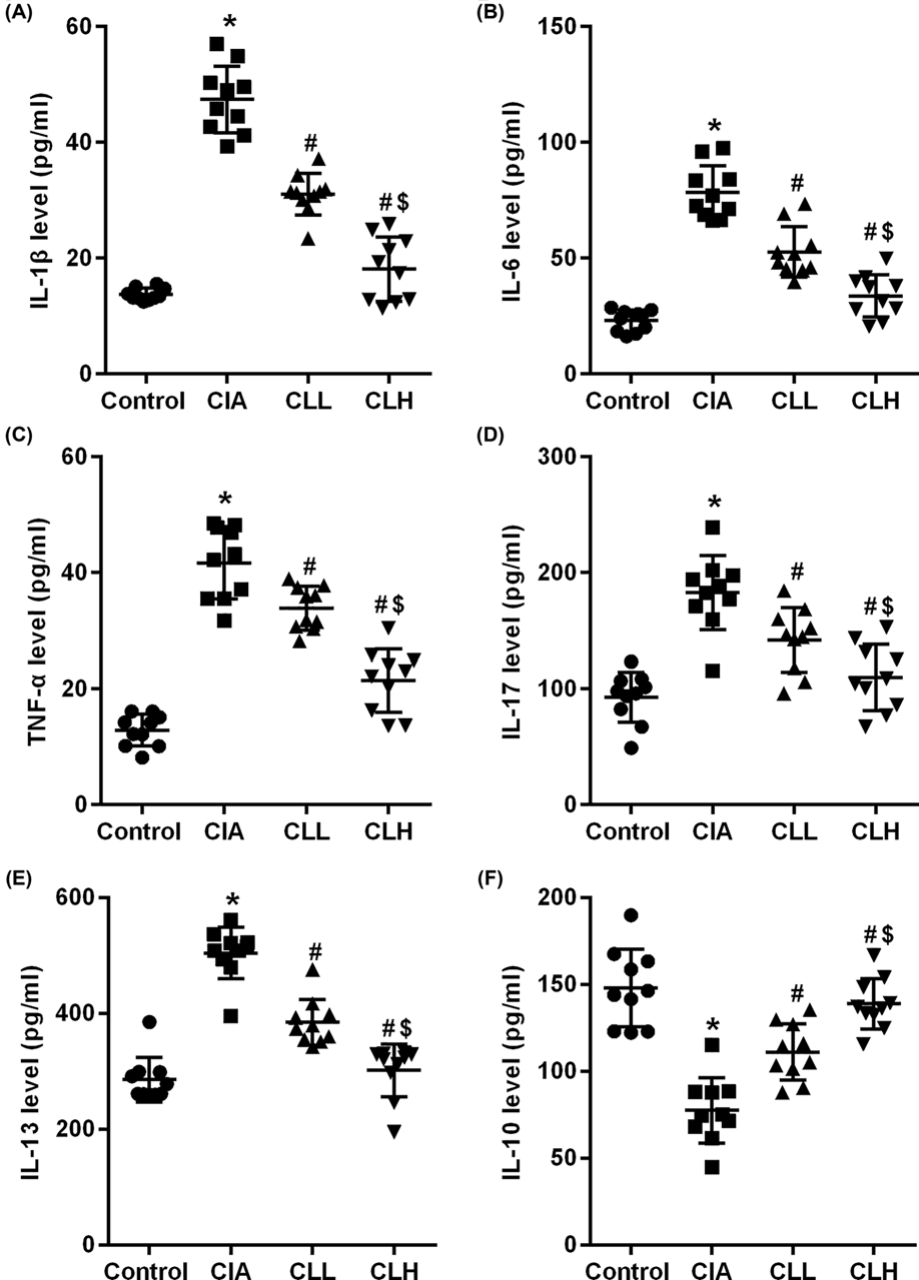
IL-38 inhibits inflammatory response in CIA rats ELISA were used to detected inflammatory response factor. (**A**) IL-1β, (**B**) IL-6, (**C**) TNF-α, (**D**) IL-17, (**E**) IL-13 and (**F**) IL-10. * vs control group, *P*<0.05; # vs CIA group; *P*<0.05; $vs CLL group, *P*<0.05.

The inflammatory factor has a certain destructive effect on articular cartilage and subchondral bone, which is also an important indicator for evaluating arthritis. In the present study, ELISA kit was used to detect the changes of inflammatory factors in rat serum. We found that the expression of inflammatory factors IL-1β (Figure 2A), IL-6 (Figure 2B), TNF-α (Figure 2C), IL-17 (Figure 2D) and IL-13 (Figure 2E) in serum of CIA rats increased while the IL-10 (Figure 2F) decreased (*P*<0.05 vs control), which suggested that collagen- induced rats cause joint damage in rats by stimulating inflammatory response in rats.

After the intervention of IL-38, the expression of IL-1β, IL-6, TNF-α, IL-17, IL-13 decreased and IL-10 increased in the CLL group (*P*<0.05 vs CIA), while in the CLH group, the expression of IL-1β, IL-6, TNF-α, IL-17 and IL-13 was significantly decreased and the IL-10 was significantly increased (*P*<0.05 vs CLL&CIA). The difference between CLL and CLH group may be related to the dose of IL-38. These results suggest that IL-38 can relieve the joint damage in rats with collagen-induced arthritis by inhibiting the inflammation.

### Effect of IL-38 on the expression of osteogenic factors in CIA rats

The synovial membrane proliferated by RA can secrete inflammatory factors and other matters that make damage to articular cartilage and subchondral bone.

**Figure 3:**
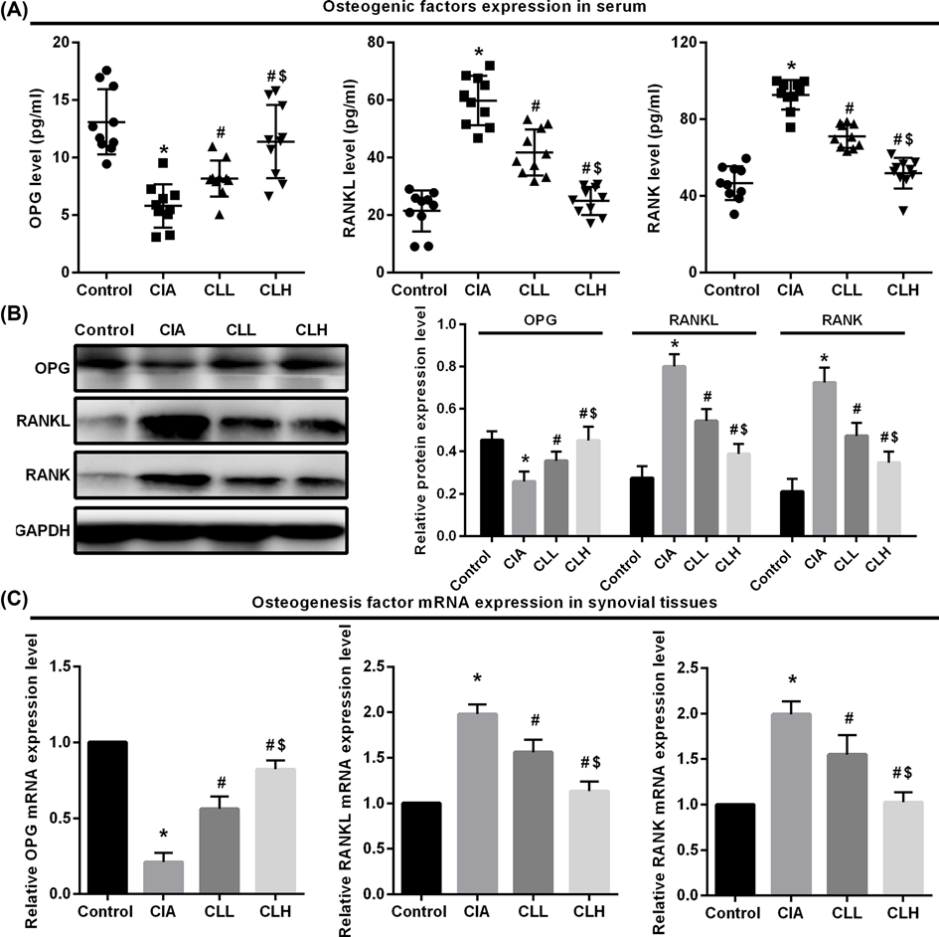
Effect of IL-38 on the expression of osteogenic factors in CIA rats ELISA, Western blot and qRT-PCR were used to detected osteogenic factor protein and mRNA expression. (**A**) ELISA, (**B**) Western blot and (**C**) qRT-PCR. * vs control group, *P*<0.05; # vs CIA group, *P*<0.05; $ vs CLL group, *P*<0.05.

RANKL/OPG/RANK pathway is an important factor connecting bone formation and bone resorption, and RANKL/OPG balance determines the level of osteoclast activation and proliferation. In order to evaluate the effect of IL-38 on bone erosion in rats, the expression of osteogenic factor in rat serum was detected by ELISA (Figure 3A). We found that after 35 days of modeling, the expression of OPG was decreased and the RANKL and RANK was increased in CIA group (*P*<0.05 vs control), suggesting that CIA rats up-regulate RANKL and RAANK expression by promoting inflammatory response, and down-regulating OPG expression leads to bone destruction in rats. After IL-38 intervention, the expression of OPG in serum of CLL group was slightly increased, and the expression of RANKL and RANK was slightly decreased (*P*<0.05 vs CIA), which suggests that IL-38 can promote the up-regulation of OPG expression in serum of CIA rats and down-regulate the expression of RANKL and RANK. By comparison, due to the factor of IL-38 dose, the expression of OPG in serum of CLH group was significantly increased, and the RANKL and RANK was significantly decreased (*P*<0.05 vs CLL&CIA).

Furthermore, Western blot (Figure 3B) and qPCR (Figure 3C) were used to detect the expression of osteogenic factors in rat synovial tissue from protein and mRNA levels. It showed that the expression of OPG in the synovial tissue of rats in CIA group was decreased, and RANKL and RANK were increased (*P*<0.05 vs control). The expression of OPG was up-regulated in the synovial tissue of rats treated with IL-38, and RANKL and RANK were down-regulated (*P*<0.05 vs CIA). It is suggested that IL-38 can inhibit the inflammatory response in rats, up-regulate the expression of OPG in CIA rats, down-regulate the expression of RANKL and RANK, and improve the joint damage in arthritic rats.

### Effect of IL-38 on the expression of CIA angiogenesis factor

**Figure 4:**
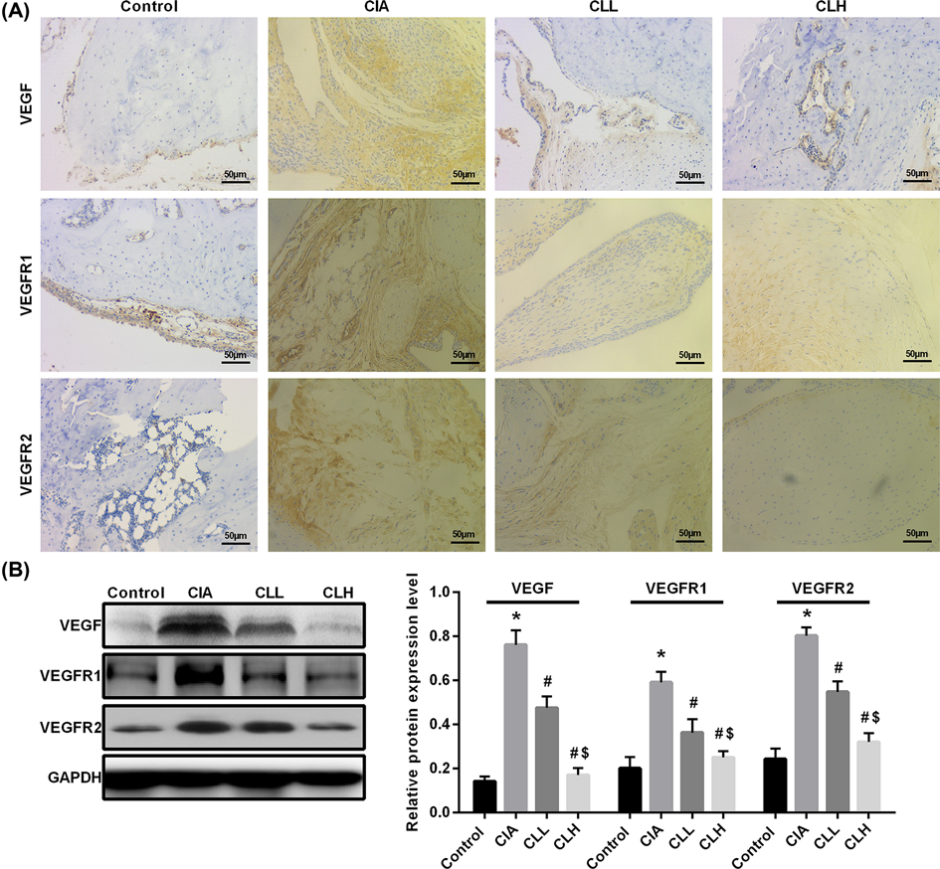
Effect of IL-38 on the expression of CIA angiogenesis factor Immunohistochemical and Western blot were used to detected angiogenic factor expression. (**A**) Immunohistochemical (scale bar = 50 μm) and (**B**) Western blot. * vs control group, *P*<0.05; # vs CIA group, *P*<0.05; $ vs CLL group, *P*<0.05.

The proliferation and infiltration of the RA synovium and the subsequent destruction of cartilage and bone are dependent on angiogenesis in the synovium. Therefore, VEGF plays an important role in promoting angiogenesis and increasing vascular permeability in the entire pathological process of RA. We detected the angiogenic factor expression of VEGF, VEGFR1 and VEGFR2 in CIA rats by Immunohistochemical (Figure 4A) and Western blot (Figure 4B), and the results showed that the expressions of VEGF, VEGFR1 and VEGFR2 increased in CIA rats (*P*<0.05 vs control), suggesting that CIA rats can promote the production of new blood vessels generate and cause joint damage in rats. After the intervention of IL-38, the expression of VEGF, VEGFR1 and VEGFR2 in CLL group slightly decreased (*P*<0.05 vs CIA), suggesting that IL-38 can inhibit the neovascularization of CIA rats by down-regulating the expression of VEGF, VEGFR1 and VEGFR2. By comparison, after the intervention of high-dose IL-38, the expression of VEGF, VEGFR1 and VEGFR2 was more pronounced in the CLH group (*P*<0.05 vs CLL&CIA). These data suggest that IL-38 can inhibit the expression of CIA angiogenesis factor by down-regulating. Angiogenesis, thereby alleviating joint damage in rats.

### IL-38 promotes the expression of SIRT1 in CIA rats

**Figure 5:**
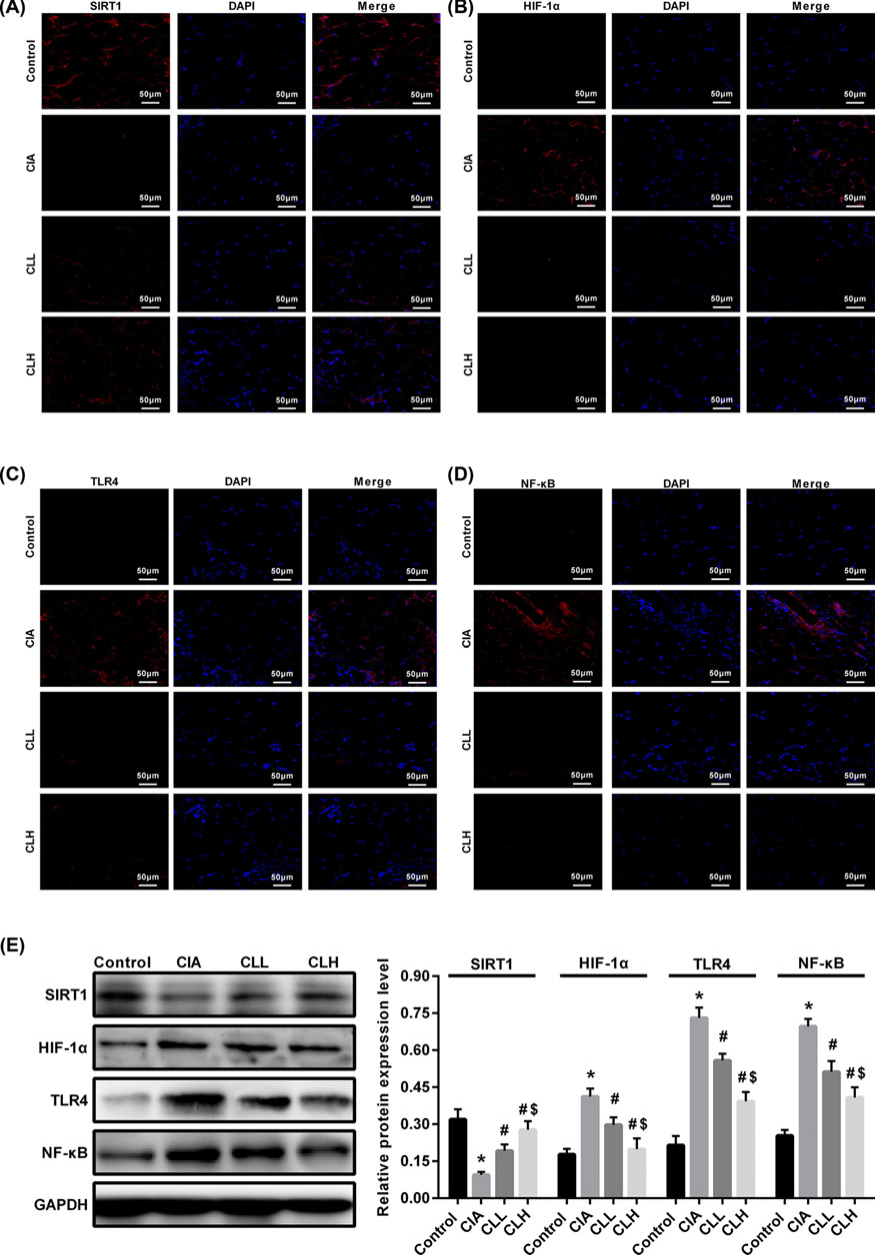
IL-38 promotes the expression of SIRT1 in CIA rats Immunofluorescence and Western blot were used to detected the expression of SIRT1/HIF-1α signaling pathway related protein. (**A–D**) Immunofluorescence (scale bar = 50 μm) and (**E**) Western blot. * vs control group, *P*<0.05; # vs CIA group, *P*<0.05; $ vs CLL group, *P*<0.05.

In the normal articular cartilage tissue, the stable expression of SIRT1 plays an important role in the regulation of cartilage homeostasis. When inflammatory factors caused by RA stimulate articular chondrocytes, the expression level of SIRT1 significantly increased. By deacetylating the NF-κB p65, the TLR4/NF-κB signaling pathway is activated to participate in the inflammatory response; meanwhile, SIRT1 can regulate HIF-1a to involve in the proliferation and differentiation of bone cells. In order to study the effect of IL-38 on SIRT1/HIF-1α pathway in synovium of CIA rats, the expression of SIRT1 (Figure 5A), HIF-1α (Figure 5B), TLR4 (Figure 5C) and NF-κB p65 (Figure 5D) was detected by immunofluorescence. The results showed that SIRT1 and HIF-α of each group were mainly located in the synovium under the articular cartilage, and the TLR4 and NF-κB p65 were obviously expressed in the cytoplasm and nucleus. The positive expression of HIF-1α, TLR4 and NF-κB p65 in the synovial group of CIA group was more obvious, and the expression of SIRT1 was less. furthermore, the positive expression of two groups of SIRT1 was higher after the treatment of IL-38, and expression of HIF-1α, TLR4 and NF-κB p65 were down-regulated. Moreover, Western blot (Figure 5E) showed that SIRT1 expression was significantly decreased in the CIA group (*P*<0.05 vs control), while HIF-1α, TLR4 and NF-κB p65 were significantly increased (*P*<0.05 vs control), suggesting that through the down-regulating of SIRT1 expression, the inflammation is triggered, resulting in joint damage in CIA rats. After the intervention of IL-38, SIRT1 was elevated in the CLL group (*P*<0.05 vs CIA), and HIF-1α, TLR4 and NF-κB p65 were decreased (*P*<0.05 vs CIA), which was affected by the IL-38 dose. By comparison, SIRT1 was significantly elevated in the CLH group (*P*<0.05 vs CIA), and HIF-1α, TLR4 and NF-κB p65 were significantly decreased (*P*<0.05 vs CIA). These results suggest that IL-38 can inhibit the inflammatory response of CIA rats to alleviate joint damage, which may be related to the up-regulation of SIRT1 expression.

### IL-38 inhibits inflammatory response in CIA rats via SIRT1/HIF-1α signaling pathway

**Figure 6:**
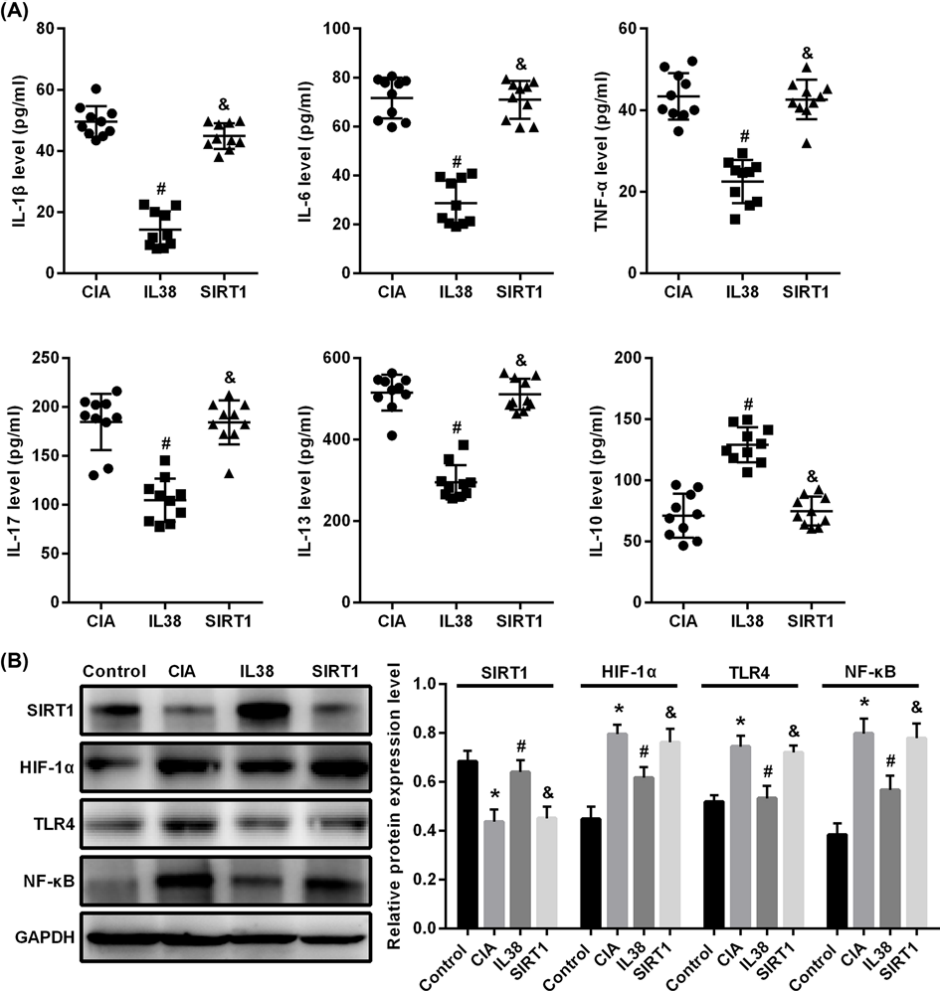
IL-38 inhibits inflammatory response in CIA rats via SIRT1/HIF-1α signaling pathway ELISA were used to detected inflammatory response factor. Western blot was used to detected the expression of SIRT1/HIF-1α signaling pathway related protein. (**A**) ELISA and (**B**) Western blot. * vs control group, *P*<0.05; # vs CIA group; *P*<0.05; $ vs CLL group, *P*<0.05.

*In vivo* experiments, we found that IL-38 can up-regulate the expression of SIRT1, down-regulate the expression of HIF-1α and inhibit the inflammatory response of CIA rats, suggesting that the mechanism of inhibiting the inflammatory response of CIA rats of IL-38 may related to the signal pathway of SIRT1/HIF-1α. In order to verify its regulatory mechanism, we established an HS cell model and added LPS to simulate CIA. Based on this, we added IL-38 and SIRT1 inhibitors respectively, and detected inflammatory factor changes in cell supernatant by ELISA (Figure 6A). The results showed that the expression of IL-1β, IL-6, TNF-α, IL-17 and IL-13 in IL-38 group decreased while IL-10 increased (*P*<0.05 vs CIA), and the inhibitory effect of inflammatory factors was attenuated after the addition of SIRT1 inhibitor (*P*<0.05, SIRT1 vs IL-38). Furthermore, after detecting the expression of SIRT1, HIF-1α, TLR4 and NF-KB p65 protein by Western blot (Figure 6B), we found that SIRT1 expression was significantly decreased in CIA group (*P*<0.05 vs control), while HIF-1α, TLR4 and NF- κB p65 were significantly increased (*P*<0.05 vs control); and the SIRT1 expression was significantly increased in IL-38 group (*P*<0.05 vs CIA group), while HIF-1α, TLR4 and NF-κB p65 were significantly decreased (*P*<0.05 vs CIA group). After adding the SIRT1 inhibition, the regulation of IL-38 to albumen was inhibited, and the expression of SIRT1, HIF-1α, TLR4 and NF-KB p65 have no significant difference from the CIA group (*P*>0.05 vs CIA group). These data indicated that IL-38 can inhibit the inflammatory response in CIA rats by up-regulating SIRT1 expression and down-regulating the HIF-1α, TLR4 and NF-KB p65 expression, while the SIRT1 inhibitors could inhibit IL-38, which may improve inflammatory response. This suggests that IL- 38 may pass SIRT1/HIF-1α signaling pathways to inhibit inflammatory responses in CIA rats and improve joint damage in rats.

## Discussion

Rheumatoid arthritis (RA) is a chronic inflammatory disease characterized by joint disease, which can lead to joint deformity and visceral involvement [17]. We believe that to inhibit the inflammation and improve the joint damage as well as the patient quality of life are the key issues in the treatment of RA. By establishing a rat model of collagen-induced arthritis, the present study aims to observe the effects of IL-38 on joint damage, inflammatory response, osteogenic factor expression, angiogenic factors and SIRT1/HIF-1α pathway-related protein expression in CIA rats. It was found that IL-38 can alleviate collagen-induced joint injury, inhibit inflammatory response, reduce osteogenic factors and angiogenic factors, and we also found that IL-38 can up-regulate SIRT1 protein expression in collagen-induced rat joints and down-regulated the expression of HIF-1α, TLR4 and NF-KB. In cell experiments, the inhibition of inflammatory response by IL-38 was inhibited by the addition of SIRT1/HIF-1α signaling pathway, which suggests that IL-38 can alleviate collagen-induced joint damage and inhibit inflammatory responses, whose machanism may be associated with regulation of the SIRT1/HIF-1α signaling pathway.

RA joint inflammation consists of synovial cells, T cells, B cells, macrophages and dendritic cells and cytokines including TNF-a, IL-1p, IL-6, IL-8, matrix metalloproteinases and IL-17, which are involved in the onset of RA [18]. Inhibiting the inflammatory response is the preferred strategy for treating the disease. IL-38 is a family member of the IL-1, which was first discovered in 2001 [19]. After the secretion, IL-38 is able to combined to the IL-36 receptor, thereby antagonizing the binding of IL-36 to its receptor to inhibit the inflammatory response and activation of the downstream signaling pathway of the IL-1 family [20]. Boutet et al. established three models of arthritis including CIA, AIA and STIA, and injected IL-38 adenovirus expression vector into the joint cavity of rats [5]. The results showed that the joint symptoms of CIA and STIA rats who intervened by IL-38 were decreased: the score index decreased, the levels of IL-17, IL-22, IL-23 and TNFα in the joint cavity decreased, and the macrophage infiltration decreased [5]. We gave IL-38 intervention based on the establishment of the CIA rat model, and the results showed that the joint swelling of the rats was relieved, the inflammatory cells of the tissues were reduced, and the proinflammatory factors IL-1β, IL-6, TNF-α, IL-17 and IL-13 in plasma were decreased. On the one hand, this confirmed that the model established by the collagen induction method can mimic RA disease; on the other hand, it suggests that IL-38 has a good inhibitory effect on inflammation in the CIA rat model.

Rheumatoid arthritis is a heterogeneous, systemic and autoimmune disease with the main clinical manifestations of symmetrical polyarthritis [21]. Due to the local osteoclast differentiation of inflammation, the RANKL/RANK signaling pathway increases the production of local osteoclasts, causing the destruction of severe bone [22]. It has been reported that OPG is a receptor that regulates the function of osteoclasts, which could combine with RANKL by high affinity to inhibit the interaction between RANKL and RANK as well as the differentiation, activation and maturation of osteoclasts and eventually inhibits the resorption of the bone [23]. The results of the present study showed that IL-38 can promote the secretion of OPG in CIA rats and inhibit the expression of RANKL and RANK. It can be seen that IL-38 plays a role in the osteogenesis system.

In the progressive destruction of bone, neovascularization is accompanied by synovial hyperplasia and inflammatory cell infiltration, which is the basis of the formation of the vasospasm and the destruction of joints [24]. VEGF and its receptors, VEGFR1 and VEGFR2, play important roles in synovial neovascularization [25]. The present study established a CIA rat model, and bone erosion and pannus appeared in the synovial tissue of rats. The expression of VEGF, VEGFR1 and VEGFR2 decreased after the addition of IL-38, suggesting that IL-38 can inhibit angiogenesis and the formation of pannus, thereby alleviating joint damage in CIA rats.

SIRT1 is involved in the deacetylation of histone and non-histone lysine residues and the regulation of target gene expression and protein activity, thereby controlling the biological properties of cellular including cell proliferation, differentiation, apoptosis, metabolism, DNA damage and stress response [26–31]. HIF-1 has the effects of hypoxia adaptation, inflammation development and tumor growth, and SIRT1 can affect apoptosis by regulating HIF-1α [32–35]. The NF-κB pathway is a central signaling node for inflammatory cytokine stimulation and lymphocyte activation [36]. SIRT1 can interact with NF-κB to reduce the transcriptional activity of NF-κB and reduce inflammation by deacetylating or disrupting its stability [37]. Matsushita et al. examining the effects of overexpression of SIRT1 in human chondrocytes on RA genes such as MMP-1, 2, 9 and 13, it was found that overexpression of SIRT1 could inhibit the up-regulation of these genes and delayed the occurrence of RA [38]. Our study found that IL-38 can promote the expression of SIRT1 in synovial tissue of CIA rats and inhibit the expression of HIF-1α, TLR4 and NF-κB p65, as well as to inhibit the inflammatory response and alleviate joint damage in CIA rats. To further confirm the regulatory mechanism of SIRT1/HIF-1α signaling pathway and IL-38 inhibition of inflammatory response in CIA rats, we found in cell experiments that the therapeutic effect of IL-38 was inhibited after the addition of SIRT1 inhibitor. Therefore, IL-38 may inhibit the inflammatory response of CIA rats and improve joint damage in rats, and the mechanism is related to the regulation of SIRT1/HIF-1α signaling pathway.

## Abbreviations

CIA: collagen-induced arthritis rats
HIF-1α: hypoxia inducible factor-1
HS: human synovial cells
RA: rheumatoid arthritis
